# Treating insomnia symptoms with medicinal cannabis: a randomized, crossover trial of the efficacy of a cannabinoid medicine compared with placebo

**DOI:** 10.1093/sleep/zsab149

**Published:** 2021-06-11

**Authors:** Jennifer H Walsh, Kathleen J Maddison, Tim Rankin, Kevin Murray, Nigel McArdle, Melissa J Ree, David R Hillman, Peter R Eastwood

**Affiliations:** 1 Centre for Sleep Science, School of Human Sciences, University of Western Australia, Crawley, WA, Australia; 2 West Australian Sleep Disorders Research Institute, Department of Pulmonary Physiology and Sleep Medicine, Sir Charles Gairdner Hospital, Nedlands, WA, Australia; 3 School of Population and Global Health, University of Western Australia, Crawley, WA, Australia; 4 School of Psychological Science, University of Western Australia, Crawley, WA, Australia; 5 Flinders Health and Medical Research Institute, College of Medicine and Public Health, Flinders University, Adelaide, SA, Australia

**Keywords:** chronic insomnia, cannabinoids, pharmacokinetics

## Abstract

**Study Objectives:**

This randomized, double-blind, placebo-controlled, crossover study was conducted to evaluate the safety and efficacy of 2 weeks of nightly sublingual cannabinoid extract (ZTL-101) in treating chronic insomnia (symptoms ≥3 months).

**Methods:**

Co-primary study endpoints were safety of the medication based on adverse event reporting and global insomnia symptoms (Insomnia Severity Index [ISI]). Secondary endpoints included: self-reported (sleep diary), actigraphy-derived, and polysomnography measurements of sleep onset latency (SOL), wake after sleep onset (WASO), total sleep time (TST), sleep efficiency (SE); and self-reported assessments of sleep quality (*s*SQ) and feeling rested upon waking. Adjusted mean differences between placebo and ZTL-101 were calculated.

**Results:**

Twenty-three of 24 randomized participants (*n* = 20 female, mean age 53 ± 9 years) completed the protocol. No serious adverse events were reported. Forty mild, nonserious, adverse events were reported (36 during ZTL-101) with all but one resolving overnight or soon after waking. Compared to placebo, ZTL-101 decreased ISI (−5.07 units [95% CI: −7.28 to −2.86]; *p* = 0.0001) and self-reported SOL (−8.45 min [95% CI: −16.33 to −0.57]; *p* = 0.04) and increased self-reported TST (64.6 min [95% CI: 41.70 to 87.46]; *p* < 0.0001), *s*SQ (0.74 units [95% CI: 0.51 to 0.97]; *p* < 0.0001), and feeling of being rested on waking (0.51 units [95% CI: 0.24 to 0.78]; *p* = 0.0007). ZTL-101 also decreased actigraphy-derived WASO (−10.2 min [95% CI: −16.2 to −4.2]; *p* = 0.002), and increased actigraphy-derived TST (33.4 min [95% CI: 23.07 to 43.76]; *p* < 0.001) and SE (2.9% [95% CI: 2.0 to 3.8]; *p* = 0.005).

**Conclusions:**

Two weeks of nightly sublingual administration of a cannabinoid extract (ZTL-101) is well tolerated and improves insomnia symptoms and sleep quality in individuals with chronic insomnia symptoms.

**Clinical Trial:**

ANZCTR; anzctr.org.au; ACTRN12618000078257.

**Statement of Significance**

Chronic insomnia is present in 6%–15% of the population and is associated with adverse health outcomes. Current pharmacologic treatment options are often unsatisfactory. Cannabinoids have been proposed as a potential new therapeutic alternative although evidence regarding their safety and efficacy is limited. In this world-first randomized double-blind placebo-controlled crossover trial of 24 participants with chronic insomnia, 2 weeks of ZTL-101 significantly decreased insomnia symptoms relative to placebo. While more adverse events were reported while taking ZTL-101 than placebo all were mild and the majority resolved soon after waking. While replication in a larger cohort is required before definitive conclusions can be drawn, these results suggest that the cannabinoid formulation ZTL-101 may provide a useful therapeutic option for individuals with chronic insomnia, at least for short-term use.

## Introduction

Chronic insomnia disorder, characterized by difficulty initiating or maintaining sleep at least 3 nights per week for at least 3 months, is present in 6%–15% of the population [1, 2], and is associated with poor health outcomes and reduced productivity. Cognitive behavioral therapy for insomnia (CBTi) is the first-line treatment, with improvement reported in approximately 60% of patients [3]. In cases where treatment is ineffective or access to CBTi is limited or delayed, then pharmacological therapies can be useful. However, adverse effects from conventional pharmacological treatments for insomnia are common [4] and include dependence, abuse potential, tolerance, daytime sedation, psychomotor impairment manifesting as falls and cognitive impairment [5] and increased risk of head injury or fracture [6]. These undesirable side-effects drive an ongoing search for alternative therapies. Cannabinoids have emerged as a possible alternative therapy for patients with insomnia who are considering therapeutic options.

Cannabis use in the United States was prohibited from 1937 until it was legalized for medical use in the late 1990s. Similar legislative changes have followed in many countries, prompting increased availability and use for medical purposes. Insomnia (or “sleep disorder”) is a common symptom for which people use cannabis [7]. However, few studies have examined the efficacy of cannabinoid formulations in treating insomnia [8], and a placebo-controlled randomized trial has yet to be undertaken. Improvements in sleep quality have been reported with delta-9-tetrahydrocannabinol (THC) and cannabidiol (CBD) alone or in combination. However, when taken in combination CBD is known to attenuate the potential psychotropic effects of THC [9] although high doses of CBD have been reported to have potential alerting properties [10]. Greater drowsiness has been reported with the addition of cannabinol (CBN) and THC than with THC alone [11].

This study therefore employed a double-blind, randomized, placebo-controlled, crossover design to evaluate the safety and efficacy of a cannabinoid formulation which included THC, CBD, and CBN (ZTL-101), for treating insomnia symptoms in patients with chronic insomnia disorder.

## Methods

### Participants

Men and women aged 25–70 years presenting with chronic insomnia, defined as self-reported difficulty initiating sleep (latency to persistent sleep >30 min) and/or maintaining sleep (>30 min awake, or waking >30 min before desired waking time) on ≥3 nights per week, for ≥3 months and an Insomnia Severity Index (ISI) score >10 [12]. Participants were recruited via email, newspaper, or television advertisement asking for volunteers to test a new medication to improve sleep quality. Only after responding to the advertisement were potential participants informed that the medication was medicinal cannabis.

Participants were excluded from the study if they: were unwilling to cease using psychotropic, CNS-depressant (confirmed at baseline with urine drug screen for opioids, amphetamine, cocaine, benzodiazepine, and cannabis), or cytochrome P450 inhibitor medications for the study duration, commencing from at least 2 weeks prior to baseline assessments; had untreated cardiovascular, metabolic, or significant psychopathologic disorders (self-reported); had other significant sleep disorders (self-reported or identified at baseline); or were participating in a behavioral therapy program to improve sleep (see Supplementary Methods for full exclusion criteria). Participants were provided with travel vouchers where required but no other compensation.

### Study design and procedures

A double-blind, randomized, placebo-controlled, crossover design study was conducted between May and December 2019 at The University of Western Australia’s Centre for Sleep Science. Zelira Therapeutics Ltd (formerly Zelda Therapeutics) sponsored the study, but the investigators independently designed it and collected, analyzed, and interpreted all data. Ethical review and approval of the study, which was in accordance with the guidelines of the International Council for Harmonisation and principles of the Declaration of Helsinki, was obtained from Bellberry Ltd (HREC2017-03-226) and The University of Western Australia (RA/4/1/9236). Informed written consent was obtained from each participant following full explanation of procedures, risks, and benefits.

**Figure 1.**
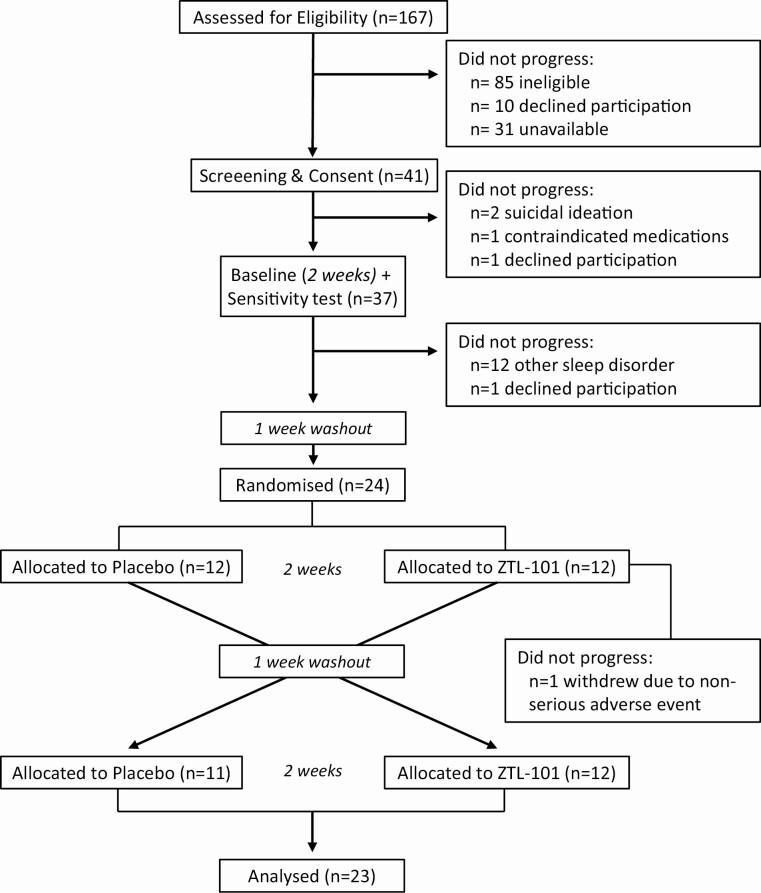
CONSORT flow diagram of participants at each phase of the trial.

Preliminary eligibility was established during a clinical interview at a screening and consent visit, which was followed by a 2-week baseline period (Figure 1) during which participants wore a wrist-based activity monitor (GT9X, ActiGraph, Pensacola, FL) and completed sleep diaries [13]. On the 14th baseline night a laboratory-based polysomnography (PSG) study (Grael, Compumedics, Sydney, Australia) was performed; participants were encouraged to attempt to sleep at their usual time and were not woken by others in the morning. The PSG montage aligned with AASM criteria [14] and studies were scored according to these criteria by a single experienced scorer who was blinded to treatment.

Potential sensitivity, including anaphalaxis, adverse cardiovascular, hallucinogenic, or paranoid responses, to a small dose of the study medication (THC/CBN/CBD; 3/0.3/0.15 mg + 0.15 mL placebo) was assessed from a 3-h observation period on the morning following this baseline PSG.

Eligible participants then underwent a 1-week washout period following which they were randomly allocated to ZTL-101 or placebo for 2 weeks (see Supplementary Methods for further detail on randomization procedure). Following a further 1-week washout period, participants crossed-over to the alternate study arm for a further 2 weeks. All participants and investigators were blinded as to whether ZTL-101 or placebo was administered. Wrist-based activity monitors were worn throughout each study arm with PSG studies performed on the 14th night of each arm.

During each of the 2-week study arms, participants sublingually self-administered 0.5 mL of ZTL-101 or placebo one, hour prior to their desired sleep time. With physician approval, participants were allowed, but not required, to double the dose (i.e. increase to two syringes of ZTL-101 or placebo = 1.0 mL sublingually) from the fourth night of each 2-week study period. Further increases in dose were not permitted, although dose decreases were. Participants were contacted daily for the first 3 days of each 2-week study period, and following dose increases, to monitor adverse events. Participants were supplied with sufficient doses for 1 week at a time and all used and unused syringes were returned to the study site to confirm doses taken.

ZTL-101 contained THC 20 mg/mL, CBN 2 mg/mL, CBD 1 mg/mL and naturally occurring terpenes, extracted from the cannabis plant, in pharmaceutical grade sunflower oil as the diluent. The investigational product was manufactured to an approved specification in a good manufacturing practice certified facility (Eurofins PROXY Laboratories, Leiden, Netherlands). The placebo contained the same terpenes, but no cannabinoids, extracted from the same cannabis plant, to match ZTL-101 as closely as possible for smell, taste, and color (Eurofins PROXY Laboratories, Leiden, Netherlands).

#### Primary outcomes

The co-primary outcome measures were: (1) frequency, type, and severity of adverse events during each of the 2-week treatment periods; and (2) global insomnia symptoms as assessed by the ISI on the 14th night of each 2-week treatment period.

#### Secondary outcomes

Measures of sleep quality and quantity were obtained from the following methods: (1) self-report from sleep diary (*s*), (2) actigraphy (*a*), and (3) PSG. Measures of sleep quality/quantity derived from each method, where possible, included sleep onset latency (SOL: time from lights off to falling asleep); total sleep time (TST: total time spent asleep overnight); wake after sleep onset (WASO: time spent awake after initially falling asleep until final out of bed time); sleep efficiency (SE: proportion of time spent asleep between the period of lights out and out of bed time); and awakening index (AI: number of awakenings per hour of sleep from lights out to out of bed time). A rating of perceived sleep quality (*s*SQ) and feeling rested/refreshed on waking was also recorded. Self-report and actigraphy measures were calculated from the mean of each of the 2-week baseline and treatment periods. The proportion of the sleep period spent in sleep stages N1, N2, N3, and REM were determined from PSG. Additional detail regarding each outcome measure is provided in Supplementary Methods.

A Maintenance of Blinding Questionnaire [15] was administered on the 14th night of each study arm to assess the success of participant blinding to treatment.

#### Pharmacokinetic study

All participants who completed both 2-week study periods were invited to an overnight study during February–March 2020 to examine the 12-h pharmacokinetic responses to a single dose (i.e. THC/CBN/CBD; 10/1/0.5 mg), and in some participants, a double dose of ZTL-101 (i.e. THC/CBN/CBD; 20/2/1 mg). Venous blood samples were drawn at 0, 1, 2, 4, 6, 8, 10, and 12 h following dosing and the plasma was analyzed for CBD, CBN, THC, and the metabolite carboxy-THC (THC-COOH) (see Supplementary Methods for further detail on pharmacokinetic study).

### Statistical analysis

#### Sample size

No previous study had reported changes in ISI, the primary outcome measure, following cannabinoid therapy for insomnia. Therefore, a sample size calculation was undertaken based on the assumption that a clinically meaningful improvement in ISI was six points [16]. Assuming a *SD* of 6 [17], alpha level of 0.05 and power of 0.9, a minimum of 13 participants were required to detect this difference. A separate sample size calculation for the secondary outcome measure of SE from PSG was also performed. In order to detect an increase in SE of 20 with a *SD* of 25 [18], power of 0.9 and alpha of 0.05, a minimum of 19 participants was required. To ensure a sufficient sample size to detect both primary and secondary outcome measures in *per protocol* analysis and to allow for a 20% attrition rate the planned sample size was 24 participants. Due to the high prevalence of obstructive sleep apnea (OSA) in the general population, approximately 40% of participants were expected to be excluded following the baseline PSG. Thus, we expected to consent a total of 40 participants, and exclude approximately 16 following consent due to the presence of OSA.

Linear mixed models were used to analyze differences in responses between the two study arms. Fixed effects of treatment (placebo vs ZTL-101) and order and period of treatment were included, along with random individual effects. Estimated differences in least squares means were calculated along with standard errors and confidence intervals. Cohen’s *d* (effect size) was calculated based on the method for paired data [19]. Whilst multiple tests were undertaken, and all results presented, the *p*-values presented have not been adjusted for multiplicity.

One participant did not complete the baseline ISI questionnaire. Screening data were imputed for this participant’s baseline data. One participant did not complete question three of the seven item ISI questionnaire on the first dosing arm (placebo). A mean of screening and baseline data (which were identical) was imputed for this question. No other data were missing.

Unless specified, data are presented as adjusted mean ± *SD* and adjusted mean difference [95% CI].

## Results

Of 167 individuals considered for inclusion, 85 were ineligible due to concomitant medications (*n* = 35), age (*n* = 23), other sleep disorders (*n* = 11), comorbidities (*n* = 4), currently participating in CBTi (*n* = 4), ISI <10 (*n* = 4), self-exclusion for unknown reason (*n* = 3), or history of falling asleep while driving (*n* = 1). In addition, 41 individuals declined participation due to lack of availability (*n* = 31) or unwillingness to participate in a pharmaceutical trial (*n* = 10) leaving 41 participants who consented for the trial. A total of 15 were excluded: 12 with significant other sleep disorders; 2 with suicidal ideation; and 1 with contraindicated medication use. A further two participants withdrew prior to initial dosing. Twenty-four participants (*n* = 20 female, mean age 53 ± 9 years) proceeded to dosing (Figure 1). One female participant withdrew after the fourth night of ZTL-101 due to nonserious adverse events (xerostomia, oral hypesthesia, swollen tongue, nausea). Twenty-three, predominantly Caucasian (*n* = 1 Asian) participants completed the full protocol (*n* = 19 female; *n* = 5 premenopausal; mean age 52 ± 9 years; mean body mass index 24.8 ± 3.6 kg/m^2^).

Of the 23 participants who completed the protocol, 12 (52%) were taking a double dose of ZTL-101 on the 14th night. Sixteen (69.5%) were taking a double dose of placebo on the 14th night. Twenty-one of 21 participants (100%) (*n* = 2 missing data) guessed that they were receiving the active medication when taking ZTL-101. “*Improvement in sleep quality*” was the reason for their guess in 17 (81%) with adverse reactions the reason in the remaining four participants. When taking the placebo, 18 of 23 participants (78%) thought they were receiving placebo. Sixteen noted “*lack of improvement in sleep quality*” as the reason for their guess that they were receiving placebo.

### Primary outcomes

#### Safety

No serious adverse events were reported. During ZTL-101 dosing, 36 nonserious adverse events possibly or likely related to ZTL-101 medication were recorded from 17 participants. Four nonserious adverse events were recorded from four participants during dosing with the placebo medication and one nonserious adverse event was reported during sensitivity testing (Table 1). All adverse events were classified as mild and had either resolved overnight or soon after waking (97.5%).

**Table 1.** Adverse events reported during ZTL-101 and placebo dosing*

Adverse event	ZTL-101 (*n* = 24)	Placebo (*n* = 23)	Sensitivity (*n* = 24)
Headache	4 (16.7)	2 (8.7)	0 (0)
Xerostomia	8 (33.3)	0 (0)	1 (4.2)
Dizziness	6 (25.0)	1 (4.2)	0 (0)
Feeling abnormal	4 (16.7)	0 (0)	0 (0)
Dry eye	2 (8.3)	0 (0)	0 (0)
Palpitations	2 (8.3)	0 (0)	0 (0)
Nausea	2 (8.3)	0 (0)	0 (0)
Ataxia	2 (8.3)	0 (0)	0 (0)
Mood variable	1 (4.2)	1 (4.2)	0 (0)
Tachyphrenia	1 (4.2)	0 (0)	0 (0)
Auditory hallucinations	1 (4.2)	0 (0)	0 (0)
Visual hallucinations	1 (4.2)	0 (0)	0 (0)
Oral hypesthesia	1 (4.2)	0 (0)	0 (0)
Swollen tongue	1 (4.2)	0 (0)	0 (0)

*All values expressed as number and (percentage) of participants.

#### Insomnia symptoms

**Figure 2.**
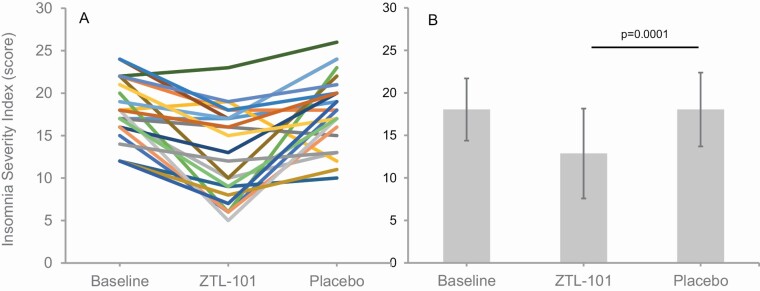
Individual (A) and group mean (±*SD*) (B) ISI scores for all participants (*n* = 23) at baseline and during treatment with ZTL-101 and placebo.

ISI scores at the end of 2 weeks of ZTL-101 were significantly lower than scores following 2 weeks of placebo (adjusted mean difference −5.1 [95% CI −2.9 to −7.3], *p* = 0.0001, *d* = 0.94; Figure 2; Table 2). ISI scores for the 12 participants who took a single dose of ZTL-101 and 11 participants who took a double dose are presented in Supplementary Results, Table S1.

**Table 2.** Group mean* measures of insomnia symptoms and sleep quantity and quality at baseline and during ZTL-101 and placebo dosing (*n* = 23)

	Baseline	ZTL-101	Placebo	*p* †	Effect size‡ *d*
Insomnia symptoms					
ISI (score)	18.0 ± 3.7	12.9 ± 5.3	18.0 ± 4.3	0.0001	0.94
Self-reported measures—sleep diary					
*s*SOL (min)	47.6 ± 38.0	38.1 ± 28.0	46.9 ± 34.4	0.0369	0.44
*s*TST (min)	300.5 ± 82.6	366.7 ± 74.5	304.1 ± 81.0	<0.001	1.27
*s*SQ§	2.5 ± 0.5	3.2 ± 0.6	2.5 ± 0.5	<0.001	1.37
Rested on waking‖	1.2 ± 0.4	1.8 ± 0.6	1.2 ± 0.5	0.0007	0.83
Actigraphy					
*a*SOL (min)	4.7 ± 1.3	4.8 ± 0.9	5.2 ± 1.9	0.2825	0.22
*a*WASO (min)	84.3 ± 23.2	71.8 ± 23.9	82.3 ± 27.7	0.0021	0.68
*a*TST (min)	398.9 ± 33.8	424.7 ± 34.7	391.2 ± 35.5	<0.001	1.44
*a*SE (%)	81.8 ± 4.3	84.8 ± 4.3	81.9 ± 4.9	<0.001	1.28
*a*AI (*n*)	24.8 ± 5.8	23.4 ± 6.0	23.6 ± 6.2	0.1236	0.03
PSG					
SOL (min)	12.3 ± 11.4	25.5 ± 22.0	25.4 ± 52.7	0.9743	0.00
WASO (min)	108.8 ± 49.5	96.2 ± 65.9	82.0 ± 51.0	0.1872	0.26
TST (min)	375.0 ± 68.4	384.3 ± 82.0	386.9 ± 72.0	0.8396	0.03
SE (%)	75.6 ± 10.6	75.8 ± 14.2	78.0 ± 12.7	0.4124	0.15
AI (*n*)	23.0 ± 8.7	22.7 ± 8.2	18.4 ± 5.6	0.0709	0.40

*Unadjusted mean ± *SD*.

^†^ *p*-value based on linear mixed model analysis of treatment responses for ZTL-101 vs placebo.

^‡^Effect size for ZTL-101 vs placebo.

^§^Rating of sleep quality 0 = very poor, 1 = poor, 2 = fair, 3 = good, 4 = very good.

^‖^Rating of feeling rested/refreshed upon waking 0 = not at all rested, 1 = slightly rested, 2 = somewhat rested, 3 = well-rested, 4 = very well-rested.

### Secondary outcomes

#### Self-reported measures from sleep diary

When taking ZTL-101 vs placebo participants perceived that they went to sleep faster (*s*SOL) (8.4 min [95% CI −16.3 to −0.6], *p* = 0.0369, *d* = 0.44), slept for longer (*s*TST) (64.6 min [95% CI 41.7 to 87.5], *p* < 0.0001, *d* = 1.27), had improved sleep quality (*sS*Q) (0.7 [95% CI 0.5 to 1.0], *p* < 0.0001, *d* = 1.37), and felt more rested/refreshed on waking (0.5 [95% CI 0.2 to 0.8], *p* = 0.0007, *d*= 0.83) (Table 2).

#### Actigraphy measures

Relative to placebo, ZTL-101 significantly decreased *a*WASO (−10.2 min [95% CI −16.2 to −4.2], *p* = 0.0021, *d* = 0.68) and increased *a*TST (33.4 min [95% CI 23.1 to 43.8], *p* < 0.001, *d* = 1.44) and *a*SE (2.9% [95% CI 2.0 to 3.8], *p* < 0.001, *d* = 1.28) (Table 2). Measurements of *a*SOL (−0.4 min [95% CI −1.2 to 0.4], *p* = 0.2825, *d* = 0.22) and *a*AI (0.1 [95% CI −1.6 to 1.8, *p* = 0.8829, *d* = 0.03]) were unchanged by ZTL-101.

#### PSG measures

Relative to placebo, ZTL-101 had minimal effect on SOL (0.42 min [95% CI −26.4 to 27.3], *p* = 0.9743, *d* = 0.00), WASO (15.1 min [95% CI −7.9 to 38.1], *p* = 0.1872, *d* = 0.26), TST (−3.5 min [95% CI −38.9 to 31.9], *p* = 0.8396, *d* = 0.03), and SE (−2.4% [95% CI −8.4 to 3.6], *p* = 0.4124, *d* = 0.15). ZTL-101 had minimal effect on the number of awakenings (4.1 [95% CI −0.4 to 8.6], *p* = 0.0709, *d* = 0.40) (Table 2), proportion of sleep stages or severity of obstructive sleep apnea or periodic limb movements (Supplementary Table S2), compared with placebo.

#### Pharmacokinetic measures

**Figure 3.**
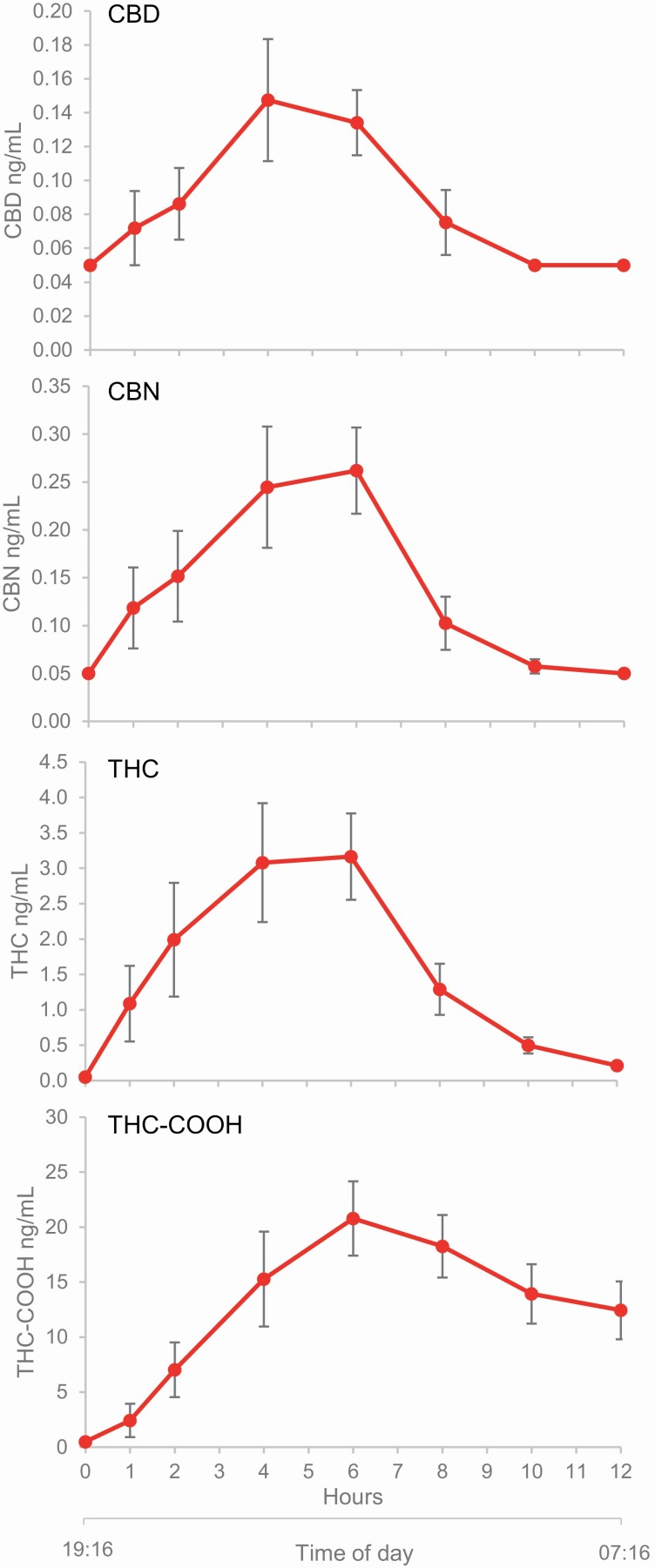
Mean plasma concentrations (±*SE*) for CBD, CBN, THC, and THC-COOH over time after administration of a single dose (*n* = 9) of ZTL-101. Hours since time of administration (h) and mean time of day (hh:mm) are shown on the *x*-axis.

Pharmacokinetic measures were obtained in all those available to return for an additional night of testing. Pharmacokinetic measures in response to a single dose of ZTL-101 were obtained in nine participants. Time at which maximal plasma concentration was reached (*T*_max_) occurred at 4–6 h for CBD, CBN, THC-COOH, and THC (Figure 3).

Pharmacokinetic measures in response to a double dose of ZTL-101 (*n* = 2) are displayed in Supplementary Figure S1 and additional pharmacokinetic parameters are shown in Supplementary Table S3.

## Discussion

This Phase 1b study demonstrated that nightly sublingual administration of a novel cannabinoid formulation for 2 weeks improved insomnia symptoms without significant adverse events in participants with chronic insomnia symptoms. This study used the ISI, a reliable, valid, and widely used instrument, to quantify perceived insomnia severity and its impact on daily function [17]. The ISI was lower while taking ZTL-101 relative to placebo. Consistent with this positive treatment response, improvements in self-reported sleep diary and objective actigraphic measures of sleep quality and quantity were also documented.

The novel formulation, ZTL-101, was well tolerated with only one participant withdrawal, due to a nonserious adverse event. Seventeen of the 24 participants experienced at least one adverse event while taking ZTL-101, with dry mouth and dizziness being most frequently reported. Although the number of adverse events is somewhat greater than that commonly reported for contemporary hypnotics [20], it is comparable to other trials using medicinal cannabis [21]. Furthermore, all adverse events were classified as mild and all but one (xerostomia, oral hypesthesia, swollen tongue, nausea in the participant who withdrew) had resolved upon waking or soon afterwards. The occurrence of dizziness and hallucinations are the most concerning and suggest caution is required in populations such as the elderly or those with psychiatric disorders. Although the occurrence of adverse events may be reduced with a more gradual dose titration [22], it is clear that further research is required to more comprehensively assess benefits and harms of medicinal cannabis use for the treatment of insomnia.

The effects of ZTL-101 on sleep were assessed from self-report, actigraphy, and PSG. Each of these methods is widely used to assess sleep, although none are without limitations in individuals with insomnia. For example, while self-reported sleep difficulty is the basis of the clinical diagnosis of insomnia, self-reported measures of total time spent asleep and time taken to fall asleep at the start of the night are known to be under and overestimated, respectively [23]. Actigraphy can objectively and unobtrusively measure sleep over multiple nights in the home environment. However, it tends to overestimate sleep time and underestimate wake time because the method designates periods of no motion as sleep, whether asleep or not. PSG is considered the “gold-standard” method of defining sleep, wake, respiration, and movement, and can therefore provide an objective measure of sleep quality, quantity, and identify other sleep disorders. It is typically performed in a laboratory or clinic on a single night and is more intrusive than actigraphy. However, its use in individuals with insomnia is limited by first night effects (i.e. patients sleeping worse or better than usual) and an inability to capture night-to-night variability in sleep behavior with a single night measurement. To enable a thorough evaluation of the impact of ZTL-101 on sleep, the present study utilized all three methods.

When taking ZTL-101 participants reported an improvement in the time taken to fall asleep, time spent asleep, and feelings of being more rested/refreshed on waking. These self-reported improvements were supported by actigraphy-derived measures of the mean total time spent asleep each night, which increased by 33.5 min; SE, which increased by 2.9%–84.8%; and the time spent awake during the night, which decreased by 10 min. The mean total time spent asleep across the 2-week period was over 7 h while taking ZTL-101, which is the recommended minimum sleep duration for adults [24], and above average for individuals of comparable age without insomnia [25]. Despite this, the average time spent awake during the night remained high at greater than 70 min, possibly reflecting a persisting tendency to disturbed sleep in individuals with chronic insomnia or that sufficient sleep had been achieved given the other improvements. Notably, the time taken to fall asleep was unchanged by ZTL-101. This has been reported in other studies [26] and could reflect a “floor effect” given the relatively short *a*SOL values at baseline in the study participants; or the inability of actigraphy to differentiate motionless wakefulness from sleep.

The improvements in self-reported and actigraphy-based measures of sleep with ZTL-101 were not seen in any PSG measure of sleep quantity or quality. This is likely due, at least in part, to the inclusion criteria being based on self-reported diagnostic criteria for insomnia [12] rather than PSG-defined insomnia, as well as the limitations of a single night PSG measure described previously. The main purpose of PSG was to identify and exclude participants with other sleep disorders such as sleep apnea and periodic limb movement disorder, which can coexist with insomnia and potentially confound interpretation of any sleep-related changes ascribed to ZTL-101. It is important to also note that ZTL-101 did not induce these sleep disorders, nor did it alter the proportion of time spent in the different sleep stages. By contrast, alteration of the proportion of sleep stages is common with many hypnotic medications; benzodiazepines, for example, are known to significantly decrease the proportion of REM sleep [27].

Insomnia is characterized by self-reported difficulties initiating or maintaining sleep [2]. As such, any globally effective pharmacological therapy for it should be capable of targeting either characteristic. To explore this, the pharmacokinetic properties of ZTL-101 overnight were determined after a standardized evening meal, thereby obtaining measurements of drug metabolism under conditions pertaining to its use in the clinical setting. Following a single dose of ZTL-101, peak plasma levels of the major constituents were reached at approximately 4–6 h. A similar profile was observed following ingestion of a double dose of ZTL-101, albeit with greater maximum plasma levels. Although it is possible that accumulation of cannabinoids might occur with dosing over multiple nights, this has not been demonstrated in dosing with THC/CBD = 21.6/20.0 mg out to 9 days. These data suggest that for patients with sleep onset insomnia dosing 2–4 h before desired bedtime might be optimal, while those with sleep maintenance insomnia should dose 1 h before desired bedtime.

## Limitations

Due to the possible risk of exacerbating preexisting conditions, individuals with a history of significant cardiovascular disease or known major psychopathology were excluded. The safety and efficacy of ZTL-101 in these populations remains to be established. Likewise, the possible drug–drug interaction between ZTL-101 and cytochrome P450 inhibitors requires further investigation. Maintenance of blinding is challenging in hypnotic [28] and cannabinoid trials [29] and this study is one of the first randomized, placebo-controlled medicinal cannabis trials to assess efficacy of blinding. The results of the blinding questionnaire suggest that despite considerable efforts to ensure ZTL-101 and placebo were similar in appearance, odor, and taste, effective blinding for a beneficial treatment effect was unable to be achieved; thus the data should be interpreted with this in mind. This is the first study to demonstrate acceptable safety and promising efficacy of a cannabinoid therapy in a randomized, double-blind, placebo-controlled manner, although it was limited to 2 weeks and included a relatively small sample of participants. Similarly, the pharmacokinetic component of the study used a subset of participants (*n* = 9), which although small is not unusual for pharmacokinetic studies [30, 31].

We undertook a multifaceted study of the influence of ZTL-101 on sleep and wakeful function, examining for a consistent trend across many measures beyond the ISI, the co-primary outcome measure. Adjustment for multiple comparisons was not performed as we did not want to artificially discount any of the exploratory analyses, which are all presented [32]. Further dedicated studies are needed to confirm the promising findings of this preliminary investigation of a novel pharmacological approach to a very common and vexing health issue.

## Conclusions

This study has demonstrated that ZTL-101, a novel cannabinoid therapy, is well tolerated and improves insomnia symptoms and sleep quality in individuals with chronic insomnia symptoms. These improvements, observed over a 2-week dosing period, are encouraging and support further investigation of ZTL-101 for the treatment of insomnia in studies with larger sample sizes.

## Supplementary Material

zsab149_suppl_Supplementary_Material

## Data Availability

The data that support the findings of this article are available from the corresponding author (JW), upon reasonable request.

## References

[1] Reynolds AC , et al Chronic insomnia disorder in Australia: a report to the Sleep Health Foundation. 2019. https://www.sleephealthfoundation.org.au/news/special-reports/chronic-insomnia-disorder-in-australia.html. Accessed July 29, 2020.

[2] Morin CM , et al Chronic insomnia. Lancet. 2012;379(9821):1129–1141. 22265700 10.1016/S0140-6736(11)60750-2

[3] Morin CM , et al Cognitive behavioral therapy, singly and combined with medication, for persistent insomnia: a randomized controlled trial. JAMA. 2009;301(19):2005–2015. 19454639 10.1001/jama.2009.682 PMC3050624

[4] Herring WJ , et al Suvorexant in patients with Insomnia: results from two 3-month randomized controlled clinical trials. Biol Psychiatry. 2016;79(2):136–148. 25526970 10.1016/j.biopsych.2014.10.003

[5] Schroeck JL , et al Review of safety and efficacy of sleep medicines in older adults. Clin Ther. 2016;38(11):2340–2372. 27751669 10.1016/j.clinthera.2016.09.010

[6] Lai MM , et al Long-term use of zolpidem increases the risk of major injury: a population-based cohort study. Mayo Clin Proc. 2014;89(5):589–594. 24684782 10.1016/j.mayocp.2014.01.021

[7] Hazekamp A , et al The medicinal use of cannabis and cannabinoids—an international cross-sectional survey on administration forms. J Psychoactive Drugs. 2013;45(3):199–210. 24175484 10.1080/02791072.2013.805976

[8] Suraev AS , et al Cannabinoid therapies in the management of sleep disorders: a systematic review of preclinical and clinical studies. Sleep Med Rev. 2020;53:101339. 32603954 10.1016/j.smrv.2020.101339

[9] Bhattacharyya S , et al Opposite effects of delta-9-tetrahydrocannabinol and cannabidiol on human brain function and psychopathology. Neuropsychopharmacology. 2010;35(3):764–774. 19924114 10.1038/npp.2009.184 PMC3055598

[10] Nicholson AN , et al Effect of Delta-9-tetrahydrocannabinol and cannabidiol on nocturnal sleep and early-morning behavior in young adults. J Clin Psychopharmacol. 2004;24(3):305–313. 15118485 10.1097/01.jcp.0000125688.05091.8f

[11] Karniol IG , et al Effects of delta9-tetrahydrocannabinol and cannabinol in man. Pharmacology. 1975;13(6):502–512. 1221432 10.1159/000136944

[12] Medicine AAoS. International Classification of Sleep Disorders. 3rd ed. Darien, IL: American Academy of Sleep Medicine; 2014. 10.5664/jcsm.4050 PMC4153104 25221449

[13] Carney CE , et al The consensus sleep diary: standardizing prospective sleep self-monitoring. Sleep. 2012;35(2):287–302. doi:10.5665/sleep.1642 22294820 PMC3250369

[14] Berry RB , et al The AASM Manual for the Scoring of Sleep and Associated Events: Rules, Terminology and Technical Specifications. Version 2.5. Darien, IL: American Academy of Sleep Medicine; 2018.

[15] Moscucci M , et al Blinding, unblinding, and the placebo effect: an analysis of patients’ guesses of treatment assignment in a double-blind clinical trial. Clin Pharmacol Ther. 1987;41(3):259–265. 3816016 10.1038/clpt.1987.26

[16] Yang M , et al Interpreting score differences in the Insomnia Severity Index: using health-related outcomes to define the minimally important difference. Curr Med Res Opin. 2009;25(10):2487–2494. 19689221 10.1185/03007990903167415

[17] Bastien CH , et al Validation of the Insomnia Severity Index as an outcome measure for insomnia research. Sleep Med. 2001;2(4):297–307. 11438246 10.1016/s1389-9457(00)00065-4

[18] Monti JM , et al Conventional and power spectrum analysis of the effects of zolpidem on sleep EEG in patients with chronic primary insomnia. Sleep. 2000;23(8):1075–1084. doi:10.1093/sleep/23.8.1g 11145322

[19] Sawilowsky SS . New effect sizes rules of thumb. J Mod Appl Stat Methods. 2009;8(2):597–599.

[20] Sateia MJ , et al Clinical practice guideline for the pharmacologic treatment of chronic insomnia in adults: an American Academy of Sleep Medicine Clinical Practice Guideline. J Clin Sleep Med. 2017;13(2):307–349. 27998379 10.5664/jcsm.6470 PMC5263087

[21] Wang T , et al Adverse effects of medical cannabinoids: a systematic review. CMAJ. 2008;178(13):1669–1678. 18559804 10.1503/cmaj.071178 PMC2413308

[22] MacCallum CA , et al Practical considerations in medical cannabis administration and dosing. Eur J Intern Med. 2018;49:12–19. 29307505 10.1016/j.ejim.2018.01.004

[23] Mercer JD , et al Insomniacs’ perception of wake instead of sleep. Sleep. 2002;25(5):564–571. doi:10.1093/sleep/25.5.559 12150323

[24] Watson NF , et al; Consensus Conference Panel. Joint consensus statement of the American Academy of Sleep Medicine and Sleep Research Society on the recommended amount of sleep for a healthy adult: methodology and discussion. Sleep. 2015;38(8):1161–1183. doi:10.5665/sleep.4886 26194576 PMC4507722

[25] Ohayon MM , et al Meta-analysis of quantitative sleep parameters from childhood to old age in healthy individuals: developing normative sleep values across the human lifespan. Sleep. 2004;27(7):1255–1273. doi:10.1093/sleep/27.7.1255 15586779

[26] Martin JL , et al Wrist actigraphy. Chest. 2011;139(6):1514–1527. 21652563 10.1378/chest.10-1872 PMC3109647

[27] Roth T , et al Effects of benzodiazepines on sleep and wakefulness. Br J Clin Pharmacol. 1981;11(Suppl 1):31S–35S. 6133532 10.1111/j.1365-2125.1981.tb01836.x PMC1401642

[28] McCall WV , et al Blinding and bias in a hypnotic clinical trial. Hum Psychopharmacol. 2021;36(1):1–5. 10.1002/hup.2757 32918323

[29] Casarett D . The Achilles heel of medical cannabis research—inadequate blinding of placebo-controlled trials. JAMA Intern Med. 2018;178(1):9–10. 29159413 10.1001/jamainternmed.2017.5308

[30] Fernandez-Trapero M , et al Pharmacokinetics of Sativex(R) in dogs: towards a potential cannabinoid-based therapy for canine disorders. Biomolecules. 2020;10(2):279–286. 10.3390/biom10020279 PMC7072526 32054131

[31] Karschner EL , et al Plasma cannabinoid pharmacokinetics following controlled oral delta9-tetrahydrocannabinol and oromucosal cannabis extract administration. Clin Chem. 2011;57(1):66–75. 21078841 10.1373/clinchem.2010.152439 PMC3717338

[32] Althouse AD . Adjust for multiple comparisons? It’s not that simple. Ann Thorac Surg. 2016;101(5):1644–1645. 27106412 10.1016/j.athoracsur.2015.11.024

