# Supplementary material for: Treating insomnia symptoms with medicinal cannabis: a randomized, crossover trial of the efficacy of a cannabinoid medicine compared with placebo
Source: Sleep. 2021 Jun 11;44(11):zsab149. doi: 10.1093/sleep/zsab149 (PMC8598183; doi:10.1093/sleep/zsab149)
Supplement: zsab149_suppl_Supplementary_Material [file zsab149_suppl_supplementary_material.docx]

**Supplementary Material**

**Treating Insomnia Symptoms with Medicinal Cannabis: A Randomized, Cross-Over Trial of the Efficacy of a Cannabinoid Medicine Compared with Placebo**

Jennifer H Walsh^1,2^

Kathleen J Maddison^1, 2^

Tim Rankin^1^

Kevin Murray^3^

Nigel McArdle^1,2^

Melissa J Ree^1,4^

David R Hillman^1,2^

Peter R Eastwood^1,2,5^

^1^ Centre for Sleep Science, School of Human Sciences, The University of Western Australia, 35 Stirling Hwy, Crawley, Western Australia 6009

^2^ West Australian Sleep Disorders Research Institute, Department of Pulmonary Physiology & Sleep Medicine, Sir Charles Gairdner Hospital, Hospital Avenue, Nedlands, Western Australia, 6009

^3^ School of Population and Global Health, The University of Western Australia, Crawley, Western Australia, 6009

^4^ School of Psychological Science, The University of Western Australia, Crawley, Western Australia, 6009

^5^ Flinders Health and Medical Research Institute, College of Medicine and Public Health, Flinders University, Adelaide, South Australia

**Corresponding Author**

Jennifer Walsh; Centre for Sleep Science, The University of Western Australia, M309, Perth, Western Australia, 6009.

Phone: +61 8 6488 8694

Email: [Jennifer.Walsh@health.wa.gov.au](mailto:Jennifer.Walsh@health.wa.gov.au)

# Supplementary Methods

### Exclusion criteria

Participants were excluded from the study if they: were currently using psychotropic, CNS-depressant or cytochrome P450 inhibitor medications; had untreated cardiovascular disease or metabolic disorders; had a known sensitivity to cannabinoids or had used cannabis in the past 2 months; had severe psychopathologic conditions including depression and anxiety; had a history of seizure, drug/alcohol abuse, suicide attempt or current suicidal ideation; had at least moderate obstructive sleep apnea (OSA) (apnea hypopnea index > 15 events/hour), untreated movement disorders (periodic limb movements (PLM) >30 events/hour or >5 events/hour with PLM arousals) or delayed sleep phase syndrome (regular wake time after 8 am); were participating in a behavioural therapy program to improve sleep; were pregnant or lactating; regularly consumed more than 2 standard alcoholic drinks and/or 400mg caffeine per day; were shift workers or athletes requiring cannabis screening or were regularly required to drive within 10 hours of dosing with study medication.

### Preliminary Trial

From the start of data collection in September 2018 until November 15, 2018, 13 participants were consented according to the procedures described above. During this time three participants had completed their 2-week treatment periods of ZTL-101 and placebo and three had started or were about to start dosing. On November 15, 2018 the investigators were informed that concurrent stability testing identified the active ingredients in ZTL-101 to be outside of the specified ranges and the trial was immediately halted. A new batch of ZTL-101 and placebo was manufactured, and the study recommenced in June 2019. No participants from the preliminary trial were included in the recommenced study (described above) and no data from any participant from the preliminary trial, including data from the three participants who completed both treatment arms, were used in the analyses from the recommenced study presented herein. One participant from the preliminary trial participated in the single dose pharmacokinetic study. Human Research Ethics approval was sought and received to recruit additional participants in order to ensure adequate power for the trial.

### Randomisation

Allocation to the first treatment arm was performed according to a computer generated (randomizer.org) randomisation of the numbers 1 to 24, which aligned with the chronological order of participant progression to dosing, into groups A (ZTL-101) and B (placebo). The labelling of ZTL-101 and placebo as A and B was performed by an independent statistician in the presence of the sponsor in the treatment packaging facility and the code was securely filed with the sponsor. All investigators and study personnel involved in the conduct and analysis of the data were blinded to the code until the final participant completed the trial and all data was analysed and database was locked.

Self-reported (*s*) measures from sleep diaries included sleep onset latency (*s*SOL: estimated time from lights off to falling asleep); total sleep time (*s*TST: estimated time spent asleep overnight) and wake after sleep onset (*s*WASO: estimated time spent awake after initially falling asleep until final out of bed time). A rating of perceived sleep quality (*s*SQ) and feeling rested/refreshed on waking was also recorded. Each measure was calculated from the mean value of each of the 2-week baseline and treatment periods.

### Secondary Outcomes

Actigraphy (*a*) measures included sleep onset latency (*a*SOL: time taken to fall asleep from bed time reported on the sleep diary); total sleep time (*a*TST: time from lights out to out of bed time reported on the sleep diary); wake after sleep onset (*a*WASO: time spent awake after initially falling asleep until final out of bed time); sleep efficiency (*a*SE; proportion of time spent asleep between the period of lights out and out of bed time) and awakening index (*a*AI: number of awakenings per hour of sleep from lights out to out of bed time). Raw actigraphy data were analysed by a single experience scorer blinded to treatment (ActiLife software, Version 6, Actigraph, Pensacola, US) according to the Cole-Kripke algorithm[1] and the methods described by Boyne et al.[2] Each actigraphy parameter was represented as the mean value from each of the 2-week baseline and treatment periods.

PSG measures included sleep onset latency (SOL; time from lights out to first epoch containing >15seconds of sleep); total sleep time (TST: total time asleep between lights out to lights on in the morning); wake after sleep onset (WASO; time spent awake from sleep onset until lights on); sleep efficiency (SE; proportion of time spent asleep between the period of lights out and lights on); and the awakening index (AI; number of awakenings from lights out to lights on). In addition, the proportion of the night spent in sleep stages N1, N2, N3 and REM were also calculated.

### Pharmacokinetic Study

Participants consenting to the additional pharmacokinetic study arrived in the evening having fasted for 6 hours and having consumed no alcohol for 24 hours. Within 1 hour of consuming a standardized high fat meal and baseline blood sampling, ZTL-101 was taken. Blood samples were drawn at 1, 2, 4, 6, 8, 10 and 12 hours post-dose. Participants were allowed to sleep during the testing period and no food was consumed for at least 8 hours after taking the medication.

Participants who were taking a double dose of ZTL-101 on night 14 of the main study were invited to attend for a second pharmacokinetic visit >1 week after the first pharmacokinetic visit.

Whole blood was drawn into 5mL Ethylenediaminetetraacetic acid (EDTA) tubes and the plasma separated by refrigerated centrifugation for 10 minutes at 2,000 x g within 60 mins of sampling. Plasma was stored at <-20°C before sending to Agilex Biolabs (SA, Australia) for analysis of CBD, CBN, THC, THC-COOH and OHTHC. Briefly, significant protein binding was removed via precipitation using trifluoroacetic acid (TFA). Solid phase extraction was then conducted followed by analysis using high performance liquid chromatography with a QTRAP5500 tandem mass spectrometry detector in negative multiple reaction monitoring mode.

Pharmacokinetic parameters were calculated for single and double doses of ZTL-101 for all participants with non-compartmental analysis using Watson LIMS software (ThermoFisher Scientific). The parameters measured included AUC_(0-t)_, t_1/2_, C_max_ and T_max_. The AUC was calculated by linear trapezoid methods.

# Supplementary Results

### Insomnia Symptoms

ISI scores while taking ZTL-101 were significantly lower than when taking placebo regardless of whether a single (adjusted mean difference -4.7 [95% CI -8.6 to -0.7], *p*=0.0253, *d*=0.7, n=12) or double dose (adjusted mean difference -5.8 [95% CI -9.3 to -2.4], *p*=0.0035, *d*=1.3, n=11) was taken (**Table S1**).

### Polysomnography measures

ZTL-101 also had minimal effect on the proportion of N1 (mean difference 1.4% [95% CI -1.4 to 4.2%], *p*=0.3155, *d*=0.21), N2 (mean difference 0.2% [95% CI -3.7 to 4.2%], *p*=8998, *d*=0.04), N3 (mean difference 1.9% [95% CI -0.5 to 4.3%], *p*=0.1116, *d*=0.35) and REM (mean difference -3.5% [95% CI -7.2 to 0.1%], *p*=0.0557, *d*=0.44) sleep compared with placebo. REM latency was significantly longer for ZTL-101 compared with placebo (mean difference 54.2min [95% CI 20.1 to 88.4min], *p*=0.0033, *d*=0.70) (**Table S2**).

### Pharmacokinetic measures

Pharmacokinetic measures in response to a single or double dose of ZTL-101 were obtained in nine and two participants, respectively. Mean plasma concentrations of CBD, CBN, THC and THC-COOH over time following administration of a single or double dose are displayed in supplement **Figure S1.**  The area under the plasma concentration curve from time 0 to the final measurable concentration (AUC_(0-t)_), elimination half-life (t_1/2_) and maximum (C_max_) and time of maximum (T_max_) measured plasma concentration over the time of measurements following administration of a single or double dose of ZTL-101 are provided in supplement **Table S3.**

# Supplementary References

1. Cole RJ, Kripke DF, Gruen W, Mullaney DJ, Gillin JC. Automatic Sleep Wake Identification from Wrist Activity. Sleep. 1992; 15 (5): 461-469.

2. Boyne K, Sherry DD, Gallagher PR, Olsen M, Brooks LJ. Accuracy of computer algorithms and the human eye in scoring actigraphy. Sleep & breathing. 2013; 17 (1): 411-417.

# List of Supplementary Figures

**Figure S1.** Mean plasma concentrations (±SE) f or (A) CBD, (B) CBN, (C) THC and (D) THC-COOH over time after administration of a single dose (red line, n=9) and double dose (black line, n=2) of ZTL-101.

Hours since time of administration (Hours) and mean time of day (hh:mm) are shown on the x-axis.

CBD, cannabidiol; CBN, cannabinol; THC, delta-9-tetrahydrocannibinol; THC-COOH, carboxy-THC.

# List of Supplementary Tables

**Table S1.** Group mean measures of insomnia symptoms in participants who took a single dose (n=11) or double dose (n=12) of ZTL-101.

**Table S2.** Group mean ± SD measures of sleep quantity at baseline and during ZTL-101 and placebo dosing (n=23).

**Table S3.** Mean pharmacokinetic parameters of CBD, CBN, THC and THC-COOH after a single and double dose of ZTL-101.

**Table S1.** Group mean measures of insomnia symptoms in participants who took a single dose (n=11) or double dose (n=12) of ZTL-101.

|  | **Baseline** | **ZTL-101** | **Placebo** | ***p*-value^a^** | **Effect size*^b^***  ***d*** |
| --- | --- | --- | --- | --- | --- |
| **Single Dose of ZTL-101 (n=11)** | | | | | |
| ISI (score) | 18.6 ± 3.3 | 14.8 ± 5.1 | 18.7 ± 5.3 | 0.0253 | 0.65 |
| **Double Dose of ZTL-101 (n=12)** | | | | | |
| ISI (score) | 17.5 ± 4.0 | 11.1 ± 5.0 | 17.4 ± 3.3 | 0.0035 | 1.26 |

ISI, insomnia severity index

^a^ p-value based on linear mixed model analysis of treatment responses for ZTL-101 vs placebo

^b^ Effect size for ZTL-101 vs placebo

**Table S2.** Group mean ± SD measures of sleep quantity at baseline and during ZTL-101 and placebo dosing (n=23).

|  | **Baseline** | **ZTL-101** | **Placebo** | ***p*-value^a^** | **Effect size*^b^***  ***d*** |
| --- | --- | --- | --- | --- | --- |
| NREM1 (% TST) | 14.4 ± 8.0 | 13.9 ± 8.3 | 12.6 ± 5.2 | 0.3155 | 0.21 |
| NREM2 (% TST) | 48.8 ± 9.6 | 49.7 ± 8.1 | 49.3 ± 7.7 | 0.8998 | 0.04 |
| NREM3 (% TST) | 16.5 ± 9.3 | 15.2 ± 6.7 | 13.3 ± 6.3 | 0.1116 | 0.35 |
| REM (% TST) | 20.3 ± 5.6 | 21.2 ± 7.4 | 24.7 ± 4.5 | 0.0557 | 0.44 |
| REM Latency (mins) | 126.5 ± 59.3 | 124.8 ± 88.1 | 70.9 ± 38.4 | 0.0033 | 0.70 |
| AHI (events/hour) | 7.6 ± 4.0 | 8.5 ± 5.6 | 8.6 ± 6.1 | 0.9941 | 0.02 |
| PLMI (events/hour) | 4.49 ± 8.0 | 5.53 ± 11.8 | 6.63 ± 9.84 | 0.5113 | 0.147 |

NREM, non-rapid eye movement; AHI, apnea hypopnea index; PLMI, periodic leg movement index.

^a^ p-value based on linear mixed model analysis of treatment responses for ZTL-101 vs placebo

^b^ Effect size for ZTL-101 vs placebo

**Table S3.** Mean pharmacokinetic parameters of CBD, CBN, THC and THC-COOH after a single and double dose of ZTL-101.

|  | **Single dose ZTL-101**  (n=9) | **Double dose ZTL-101**  (n=2) |
| --- | --- | --- |
| **CBD** | | |
| AUC_(0-t)_ (ηg/mL x hour) | 1.05 (0.74-1.53) | 1.45 (1.20-1.75) |
| t_1/2_ (hours) | 3.33 (1.87-7.14) | 2.59 (2.38-2.80) |
| C_max_ (ηg/mL) | 0.19 (0.12-0.29) | 0.26 (0.23-0.30) |
| T_max_ (hours) | 4 (4-8) | 5 (4-6) |
| **CBN** | | |
| AUC_(0-t)_ (ηg/mL x hour) | 1.69 (0.98-2.82) | 3.40 (2.66-4.35) |
| t_1/2_ (hours) | 2.43 (1.73-4.98) | 1.87 (1.64-2.1 |
| C_max_ (ηg/mL) | 0.36 (0.20-0.58) | 0.64 (0.55-0.74) |
| T_max_ (hours) | 4 (1-6) | 5 (4-6) |
| **THC** | | |
| AUC_(0-t)_ (ηg/mL x hour) | 19.50 (14.08-32.78) | 51.01 (35.04-74.42) |
| t_1/2_ (hours) | 1.56 (1.21-2.12) | 1.79 (1.46-2.11) |
| C_max_ (ηg/mL) | 4.69 (3.20-6.98) | 10.60 (8.2-13.70) |
| T_max_ (hours) | 4 (1-6) | 5 (4-6) |
| **THC-COOH** | | |
| AUC_(0-t)_ (ηg/mL x hour) | 145.82 (67.85-362.75) | 282.61 (213.32-374.41) |
| t_1/2_ (hours) | 5.29 (3.07-22.87) | 4.90 (3.64-6.20) |
| C_max_ (ηg/mL) | 22.93 (14.30-40.10) | 40.96 (27.60-60.80) |
| T_max_ (hours) | 4 (4-8) | 8 (8-8) |

AUC_(0-t)_, area under the plasma concentration curve from time 0 to the final measurable concentration; t_1/2_, elimination half-life; C_max_, maximum measured plasma concentration over the time of measurements; T_max_, time of the maximum measured plasma concentration; CBD, cannabidiol; CBN, cannabinol; THC, delta-9-tetrahydrocannibinol; THC-COOH, carboxy-THC.

Data are presented as mean (range) for AUC_(0-t)_ and C_max_; and median (range) for t_1/2_ and T_max_.

**
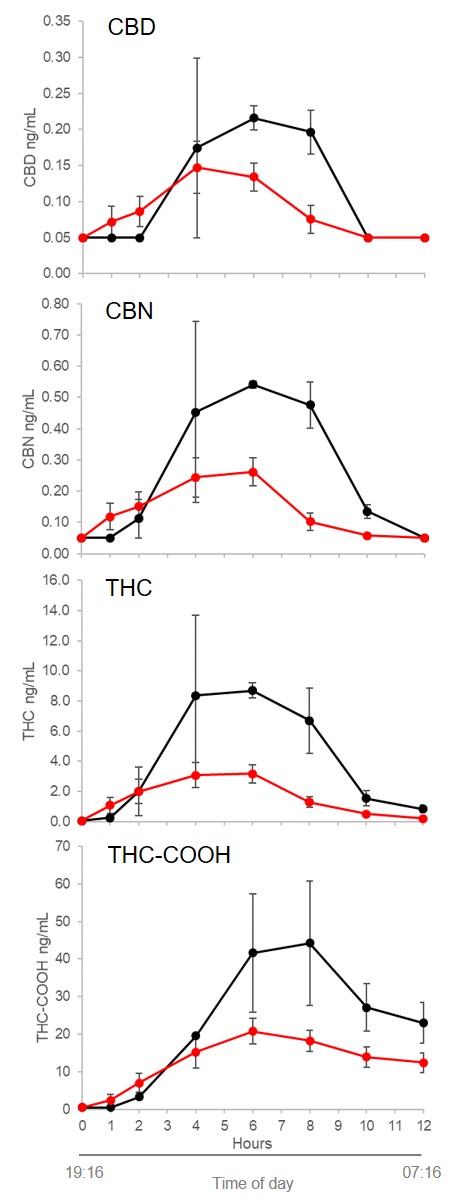
**

**Figure S1.** Mean plasma concentrations (±SE) f or (A) CBD, (B) CBN, (C) THC and (D) THC-COOH over time after administration of a single dose (red line, n=9) and double dose (black line, n=2) of ZTL-101.

Hours since time of administration (Hours) and mean time of day (hh:mm) are shown on the x-axis.

CBD, cannabidiol; CBN, cannabinol; THC, delta-9-tetrahydrocannibinol; THC-COOH, carboxy-THC.
